# A Deep Learning Framework for Signal Detection and Modulation Classification

**DOI:** 10.3390/s19184042

**Published:** 2019-09-19

**Authors:** Xiong Zha, Hua Peng, Xin Qin, Guang Li, Sihan Yang

**Affiliations:** PLA Strategic Support Force Information Engineering University, Zhengzhou 450001, Henan, Chinaqinxin_0920@163.com (X.Q.); GL_for_study@outlook.com (G.L.);

**Keywords:** deep learning, signal detection, modulation classification, the single shot multibox detector networks, the multi-inputs convolutional neural networks

## Abstract

Deep learning (DL) is a powerful technique which has achieved great success in many applications. However, its usage in communication systems has not been well explored. This paper investigates algorithms for multi-signals detection and modulation classification, which are significant in many communication systems. In this work, a DL framework for multi-signals detection and modulation recognition is proposed. Compared to some existing methods, the signal modulation format, center frequency, and start-stop time can be obtained from the proposed scheme. Furthermore, two types of networks are built: (1) Single shot multibox detector (SSD) networks for signal detection and (2) multi-inputs convolutional neural networks (CNNs) for modulation recognition. Additionally, the importance of signal representation to different tasks is investigated. Experimental results demonstrate that the DL framework is capable of detecting and recognizing signals. And compared to the traditional methods and other deep network techniques, the current built DL framework can achieve better performance.

## 1. Introduction

Cognitive radio (CR) [1,2,3] has been used to refer to radio devices that are capable of learning and adapting to their environment. Due to the increasing requirements for wireless bandwidth of radio spectrum, automatic signal detection and modulation recognition techniques are indispensable. It can help users to identify the modulation format and estimate signal parameters within operating bands, which will benefit communication reconfiguration and electromagnetic environment analysis. Besides, it is widely used in both military and civilian applications, which have attracted much attention in the past decades [4,5,6,7].

Multi-signals detection is a task to detect the existing signals in a specific wideband, which is one of the essential components of CR. The most significant difference between signal and non-signal is energy. Hence, many wideband multi-signals detection algorithms are based on energy detector (ED). Some threshold-based wideband signal detection methods, such as [8,9,10,11,12,13], reduce the probability of false alarm or missed alarm. However, these methods are sensitive to noise changes and challenging to ensure the detection accuracy of all detection scenarios. Therefore, many non-threshold-based detection algorithms have been proposed [14,15,16,17]. However, these algorithms have high computational complexity, which results in poor online detection performance.

For automatic modulation recognition, algorithms based on signal phase, frequency, and amplitude have been widely used [18]. However, these algorithms are significantly affected by noise, and the performance can be substantially degraded in low SNR condition. High-order statistical-based algorithms [19,20,21,22], such as signal high-order cumulants and cyclic spectrum, have excellent anti-noise performance. The computational complexity of these methods is relatively low, but the selection of features relies too much on expert experience. It is difficult to obtain features that can adapt to non-ideal conditions. In particular, it is challenging to set the decision threshold when there are plenty of modulation formats to be classified.

Deep Learning (DL) techniques [23,24] have made outstanding achievements in Computer Vision [25,26] (CV) and Natural Language Processing [27,28] (NLP) for their strong self-learning ability. Recently, more and more researchers use DL techniques to solve signal processing problems. For signal detection, many DL-based methods, such as [29,30,31], detect signals in narrowband environment. These methods only detect the existing of signal, but can not estimate the relevant parameters. Therefore, developing a technique leverages deep learning to detect signal efficiently and effectively is still a challenging problem. For DL-based modulation classification, there has been some reported work, including [32,33,34,35,36]. For example, some researchers used the signal IQ waveform as data representation and learned the sample using CNNs [32,33,34]. Other researchers focused on developing methods to represent modulated signals in data formats for CNNs. Among these methods, constellation-based algorithms [35,36] have been widely utilized, where signal prior knowledge is fully considered.

In this study, DL techniques are fully utilized in multi-signals detection and modulation recognition. For multi-signals detection, we use the deep learning target detection network to detect the location of each signal. In our initial research, the used model is SSD networks, which is a relatively advanced target detection network. Furthermore, we use the time-frequency spectrum as the signal characteristic expression. Due to the time-frequency characteristic of the M-ary Frequency Shift Keying (MFSK) format signals, we can identify the modulation format while the signal is detected. Meanwhile, for M-ary Phase Shift Keying (MPSK), M-ary Amplitude Phase Shift Keying (MAPSK), and M-ary Quadrature Amplitude Modulation (MQAM) signal, the difference in the time-frequency spectrum is not sufficient to identify the signal modulation. Therefore, during the signal detection procession, we identify them in the same format, and only detect the signal presence or absence. Through the signal detection network, we can roughly get the signal carrier frequency and start-stop time. After that, we use a series of traditional methods to convert these signals from the wideband into the baseband. To recognize MPSK, MAPSK, and MQAM signals, a multi-inputs CNNs is designed. Moreover, we adopt the signal vector diagram and eye diagram as the network inputs, which are more robust than in-phase and quadrature (IQ) waveform data and constellation diagram.

This paper addresses the topic of DL based multi-signals detection and modulation classification. The main contributions of this paper are summarized as follows: (1) We propose a relatively complete DL framework for signal detection and modulation recognition, which is more intelligent than traditional algorithms. (2) We establish different signal representation schemes for several tasks, which facilitate the use of the built DL framework for detection and classification. (3) We propose a multi-inputs CNNs model to extract and map the features from different dimensions.

The rest of this paper is presented as follow. In Section 2, we offer a detail introduction to the signal model and the dataset generation. Section 3 shows the DL framework for signal detection and modulation recognition. Section 4 confirms our initial experiment result from different aspects. Finally, our conclusions and directions for further research are given in Section 5.

## 2. Communication Signal Description and Dataset Generation 

In realistic communication processing, the signal may be distorted by the effect of non-linear amplifier and channel. In actual situation, the received signal in the communication system can be expressed as:

(1) $$r(t)=\int_{\tau =0}^{\tau_{0}}s(n_{clk}(t-\tau ))h(\tau )d\tau +n_{add}(t)$$

where s(t) is the transmission signal, $n_{Clk}(t)$ is timing deviation, h(t) represents the transmitted wireless channel, $n_{add}(t)$ is additive white Gaussian noise.

In this section, we will describe different modulated signals and their sample representation for our DL framework. We will also explain the reason why we use it and the method we enhance it.

### 2.1. Modulation Signal Description

For any digital modulation signal, the transmission signal can be presented as

(2) $$s(t)=\sum_{n}a_{n}e^{j(w_{n}t+\phi )}g(t-nT_{b})$$

where $w_{n}$ is the signal angular frequency, ϕ is the carrier initial phase, $T_{b}$ is the symbol period, $a_{n}$ is the symbol sequence, g(t) is the shaping filter.

For MFSK signal, it can be presented as

(3) $$a_{n}=1\;\;,w_{n}=w_{0}+\frac{2\pi }{M}i,\;i=0,1,\ldots ,M-1$$

For MPSK signal, it can be presented as

(4) $$a_{n}=e^{j2\pi i/M},\;\;\;\;\;\;\;\;i=0,1,\ldots ,M-1,w_{n}=w_{0}$$

For MQAM signal, it can be presented as

(5) $$\begin{array}{c} a_{n}=I_{n}+jQ_{n}\\ I_{n},Q_{n}=2i-\frac{M}{4}+1,i=0,1,\ldots ,\frac{M}{4}-1,w_{n}=w_{0} \end{array}\}$$

MAPSK constellations are robust against nonlinear channels due to their lower peak-to-average power ratio (PAPR), compared with QAM constellations. Therefore, APSK was mainly employed and optimized over nonlinear satellite channels during the last two decades. As recommended in DVB-S2 [37], it can be presented as:

(6) $$a_{n}=r_{k}\exp\left[j\left(\frac{2\pi }{n_{k}}i_{k}+\theta_{k}\right)\right]$$

where $r_{k}$ is the radius of the *k*th circle, $n_{k}$ is the number of constellations in *k*th circle, $i_{k}$ is the ordinal number of constellation points in the *k*th circle, $\theta_{k}$ is the initial phase of the *k*th circle.

### 2.2. Signal Time-Frequency Description

For multi-signals detection task, we use the wideband signal time-frequency spectrum as the neural network input. To prove the feasibility of this method, we theoretically prove the time-frequency visual characteristic of each modulation. Here, we use the short-time Fourier transform [38] (STFT) to analyze the signal time-frequency characteristic.

#### 2.2.1. MFSK Signal Time-frequency Description

The STFT of MFSK signal can be expressed as

(7) $$STFT_{s_{FSK}}(t,w)=\int_{-\infty }^{+\infty }[s_{FSK}(\tau )\gamma^{*}(\tau -t)]e^{-j\omega \tau }d\tau \;\;\;\;=\int_{-\infty }^{+\infty }\left[\sum_{k=-\infty }^{+\infty }Ag(\tau -kT_{b})e^{j(\omega_{k}\tau +\phi_{k})}\gamma^{*}(\tau -t)\right]e^{-j\omega \tau }d\tau$$

where γ(t) is the window function, whose duration is T. When γ(t) is in a symbol duration, Equation (7) can be simplified as

(8) $$\begin{array}{ll} STFT_{s_{FSK}}(t,w)= & \int_{-T/2}^{T/2}Ae^{j(w_{k}(\tau +t)+\phi_{k})}e^{-jw(\tau +t)}d\tau =ATe^{-jwt}e^{j(w_{k}t+\phi_{k})}Sa(\frac{w-w_{k}}{2}T)\;\;\;,\\ & kT_{b}+T/2<t<(k+1)T_{b}-T/2,k=0,1,2,\ldots \end{array}$$

where Sa(w)=sin(w)/w. When γ(t) spans two symbols, Equation (7) can be simplified as

(9) $$\begin{array}{ll} STFT_{s_{FSK}}(t,w) & =\int_{-T/2}^{d}Ae^{j(w_{k}(\tau +t)+\phi_{k})}e^{-jw(\tau +t)}d\tau +\int_{d}^{T/2}Ae^{j(w_{k+1}(\tau +t)+\phi_{k+1})}e^{-jw(\tau +t)}d\tau =\\ & =Ae^{j((w_{k}-w)t+\phi_{k})}\int_{-T/2}^{d}e^{-j(w-w_{k})\tau }d\tau +Ae^{j((w_{k+1}-w)t+\phi_{k+1})}\int_{d}^{T/2}e^{-j(w-w_{k+1})\tau }d\tau =\\ & =A\frac{T+2d}{2}e^{j((w_{k}-w)t+\phi_{k})}e^{\frac{j(w-w_{k})(T-2d)}{4}}Sa(\frac{\left(w-w_{k})(T+2d\right)}{4})+\\ & +A\frac{T-2d}{2}e^{j((w_{k+1}-w)t+\phi_{k+1})}e^{\frac{j(w-w_{k+1})(T+2d)}{4}}Sa(\frac{\left(w-w_{k+1})(T-2d\right)}{4}),\\ (k+1)T_{b} & -T/2<t<(k+1)T_{b}+T/2,d=(k+1)T_{b}-t,k=0,1,2,\ldots \end{array}$$

where $w_{k+1}$ is the carrier angular frequency of the k+1-th symbol. If $w_{k+1}=w_{k}$, it indicates that the carrier angular frequency does not jump, so Equation (8) is same as Equation (9). We take the modulus square of Equation (8). The result can be expressed as

(10) $$\begin{array}{c} SPEC_{s_{FSK}}(t,w)={\left|STFT_{s_{FSK}}(t,w)\right|}^{2}=A^{2}T^{2}Sa^{2}(\frac{w-w_{k}}{2}T)\;\;\;,\\ kT_{b}+T/2<t<(k+1)T_{b}-T/2,k=0,1,2,\ldots \end{array}$$

And for Equation (9), it can be expressed as:

(11) $$\begin{array}{c} SPEC_{s_{FSK}}(t,w_{k})\approx \frac{A^{2}{\left(T+2d\right)}^{2}}{4}\leq A^{2}T^{2}\;\;\;\;\;,-T/2<d<T/2,\\ (k+1)T_{b}-T/2<t<(k+1)T_{b}+T/2\;\;,k=0,1,2,\ldots \;\;\;\;\;\;\; \end{array}$$

Obviously, the value of $SPEC_{s_{FSK}}(t,w_{k})$ will increase as the increase of jumping time d. The energy decreases gradually as γ(t) slips away from the symbol. So when d=T/2, the window is completely within one symbol, and the maximum value is obtained.

(12) $$\begin{array}{c} SPEC_{s_{FSK}}{\left(t,w_{k}\right)}_{\max}=A^{2}T^{2},\\ \;\;(k+1)T_{b}-T/2<t<(k+1)T_{b}+T/2,k=0,1,2,\ldots \end{array}$$

When d=−T/2, the window completely spans to next symbol, and the minimum value is obtained

(13) $$\begin{array}{c} SPEC_{s_{FSK}}{\left(t,w_{k}\right)}_{\min}=0\;\;,\;\;\\ \;\;\;(k+1)T_{b}-T/2<t<(k+1)T_{b}+T/2,k=0,1,2,\ldots \end{array}$$

From our analysis, we can easily get the characteristics of FSK modulation: (1) There will be sharp brightness changes in the time-frequency image at the frequency change moment. (2) The signal modulation number *M* and frequency spacing are important parameters for the MFSK time-frequency characteristics, which determine the value of $w_{k}$.

#### 2.2.2. Amplitude–Phase Modulation Signal Time-frequency Description

For MPSK, MAPSK, and MQAM signal, since they all belong to amplitude-phase modulation, the derivation processing of the signal time-frequency characteristics is the same as MPSK. Hence, we specify the time-frequency characteristics of the MPSK signal, and the STFT can be expressed as:

(14) $$\begin{array}{ll} STFT_{s_{PSK}}(t,w) & =\int_{-\infty }^{+\infty }[s_{PSK}(\tau )\gamma^{*}(\tau -t)]e^{-jw\tau }d\tau =\\ & =\int_{-\infty }^{+\infty }\left[\sum_{k=-\infty }^{+\infty }Ag(\tau -kT_{b})e^{j(w_{c}\tau +\phi_{c}+\phi_{k})}\gamma^{*}(\tau -t)\right]e^{-jw\tau }d\tau \end{array}$$

As the derivation of MFSK signal time-frequency characteristics, when γ(t) is in a symbol duration, the Equation (14) can be simplified as:

(15) $$\begin{array}{ll} STFT_{s_{PSK}}(t,w)= & \int_{-T/2}^{T/2}Ae^{j(w_{c}(\tau +t)+\phi_{c}+\phi_{k})}e^{-jw(\tau +t)}d\tau =ATe^{-jwt}e^{j(w_{c}t+\phi_{c}+\phi_{k})}Sa(\frac{w-w_{c}}{2}T),\\ & kT_{b}+T/2<t<(k+1)T_{b}-T/2,k=0,1,2,\ldots \end{array}$$

When γ(t) spans two symbols, the Equation (14) can be simplified as:

(16) $$\begin{array}{l} STFT_{s_{PSK}}(t,w)=\int_{-T/2}^{d}Ae^{j(w_{c}(\tau +t)+\phi_{c}+\phi_{k})}e^{-jw(\tau +t)}d\tau +\int_{d}^{T/2}Ae^{j(w_{c}(\tau +t)+\phi_{c}+\phi_{k+1})}e^{-jw(\tau +t)}d\tau =\\ \;\;\;\;\;\;\;\;\;\;\;\;\;\;\;\;\;\;\;\;\;\;\;\;\;\;=Ae^{j((w_{c}-w)t+\phi_{c}+\phi_{k})}\int_{-T/2}^{d}e^{-j(w-w_{c})\tau }d\tau +Ae^{j((w_{c}-w)t+\phi_{c}+\phi_{k+1})}\int_{d}^{T/2}e^{-j(w-w_{c})\tau }d\tau =\\ \;\;\;\;\;\;\;\;\;\;\;\;\;\;\;\;\;\;\;\;\;\;\;\;\;\;=A\frac{T+2d}{2}e^{j((w_{c}-w)t+\phi_{c}+\phi_{k})}e^{\frac{j(w-w_{c})(T-2d)}{4}}Sa(\frac{\left(w-w_{c})(T+2d\right)}{4})+\\ \;\;\;\;\;\;\;\;\;\;\;\;\;\;\;\;\;\;\;\;\;\;\;\;\;\;+\;\;A\frac{T-2d}{2}e^{j((w_{c}-w)t+\phi_{c}+\phi_{k+1})}e^{\frac{j(w-w_{c})(T+2d)}{4}}Sa(\frac{\left(w-w_{c})(T-2d\right)}{4}),\\ \;\;\;\;\;\;\;\;\;\;(k+1)T_{b}-T/2<t<(k+1)T_{b}+T/2,d=(k+1)T_{b}-t,k=0,1,2,\ldots \end{array}$$

where $\phi_{k+1}$ is the phase of the k+1-th symbol. And if $\phi_{k+1}=\phi_{k}$, Equation (15) is equal to Equation (16). We take the modulus square of (15), and the result can be expressed as:

(17) $$\begin{array}{r} SPEC_{s_{PSK}}(t,w)={\left|STFT_{s_{PSK}}(t,w)\right|}^{2}=A^{2}T^{2}Sa^{2}(\frac{w-w_{c}}{2}T),\\ kT_{b}+T/2<t<(k+1)T_{b}-T/2,k=0,1,2,\ldots \end{array}$$

And for (16), it can be expressed as:

(18) $$\begin{array}{c} SPEC_{s_{PSK}}(t,w_{c})=\frac{A^{2}T^{2}}{2}(1+\cos(\phi_{k}-\phi_{k+1}))+2A^{2}d^{2}(1-\cos(\phi_{k}-\phi_{k+1}))\leq A^{2}T^{2},\\ (k+1)T_{b}-T/2<t<(k+1)T_{b}+T/2,d=(k+1)T_{b}-t,k=0,1,2,\ldots \end{array}$$

We take the partial derivative for Equation (18):

(19) $$\begin{array}{c} \frac{\partial SPEC_{s_{PSK}}(t,w_{c})}{\partial d}=4A^{2}d(1-\cos(\phi_{k}-\phi_{k+1})),-T/2<d<T/2,\\ (k+1)T_{b}-T/2<t<(k+1)T_{b}+T/2,k=0,1,2,\ldots \end{array}$$

From Equation (19), we can easily learn that $SPEC_{s_{PSK}}(t,w_{c})$ get the minimum value when $\phi_{k+1}$ = $\phi_{k}$ or d = 0. But the minimum value is much greater than 0, which is greatly different for the MFSK signal.

(20) $$\begin{array}{c} SPEC_{s_{PSK}}{\left(t,w_{c}\right)}_{\min}=\frac{A^{2}T^{2}}{2}(1+\cos(\phi_{k}-\phi_{k+1}))\gg 0,\\ (k+1)T_{b}-T/2<t<(k+1)T_{b}+T/2,k=0,1,2,\ldots \end{array}$$

**Figure 1:**
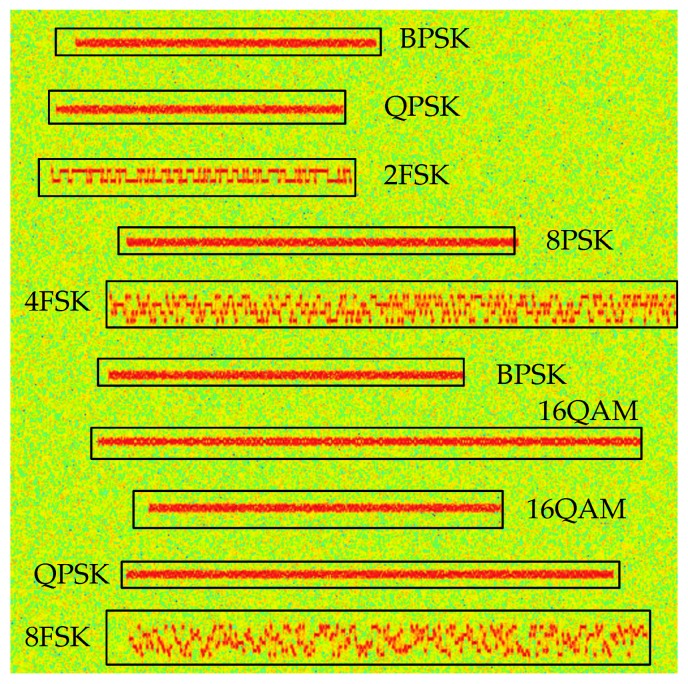
Different modulation signals in the wideband.

Hence, for MPSK signal, there is only one wide frequency band in the time-frequency diagram, and the brightness fluctuation appears in a small range, which is different from MFSK. And from derivation processing, we can know that the MPSK time-frequency characteristics are less affected by M, so it is hard to distinguish PSK signals with different M. Figure 1 presents different modulation signals in the wideband.

### 2.3. Signal Eye Diagram and Vector Diagram Description

**Figure 2:**
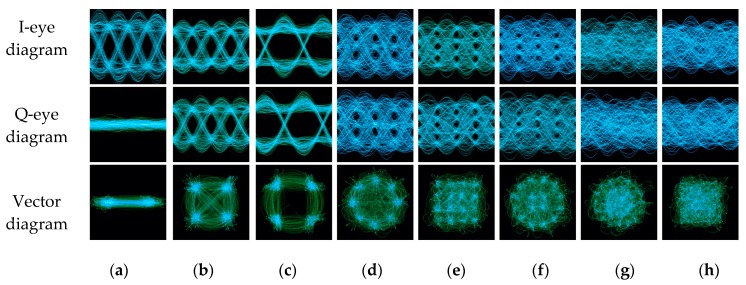
The eye diagram and vector diagram of different modulation signals in 15dB (**a**) BPSK; (**b**) QPSK; (**c**) OQPSK; (**d**) 8PSK; (**e**) 16QAM; (**f**) 16APSK; (**g**) 32APSK; (**h**) 64QAM.

The function of the eye diagram is to observe the baseband signal waveform by an oscilloscope. Through the eye-diagram, we can adjust the receiver filter to improve system performance. Besides, due to the characteristics of the modulated signal itself, different modulation modes have apparent visual differences in the eye diagram. As shown in Figure 2, because of the different modulation scales, there are different eye numbers in each eye diagram. For OQPSK, since the two orthogonal signals stagger for half a symbol period, the eye-opening position is always staggered, while other modulated signals always appear at the same time.

By reconstructing the signal IQ waveforms in the corresponding time, the signal vector diagram shows the symbol trajectory. From its formation mechanism, it is similar to the signal constellation diagram. However, unlike the constellation diagram, the vector diagram can reflect the signal phase information. For example, it can easily distinguish QPSK from OQPSK with the same initial phase, because there is no 180° phase shift in OQPSK, while it exists in QPSK. Meanwhile, compared with the constellation diagram, the vector diagram is more convenient to obtain and requires less prior information.

### 2.4. The Generation Processing of the Dataset

**Figure 3:**
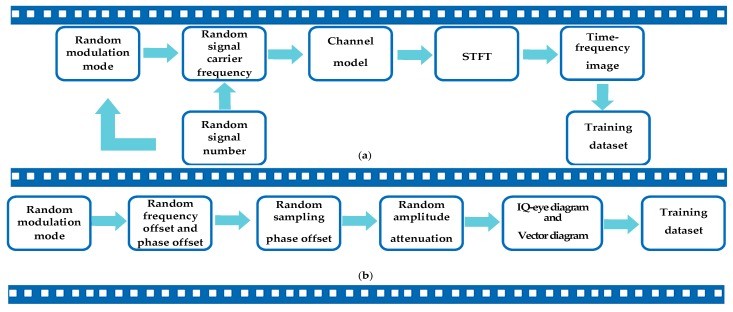
The generation processing of the dataset. (**a**) dataset for signal detection; (**b**) dataset for modulation recognition.

Figure 3 presents the processing of our dataset construction and annotation. To make samples more diverse, we set sampling phase offset, frequency offset, phase offset, and amplitude attenuation in sample generation processing.

For signal detection, we need to determine the reconnaissance frequency range and set the signal number in the wideband at first. We set different frequency offset for each signal, and ensure that the signals do not overlap in the frequency domain. Then we perform the STFT on the wideband. Not only we record the modulation format of each signal, but also record the start-stop time, carrier frequency, and bandwidth. Then we convert them into the coordinates on time-frequency image, which are the label information for the network.

**Figure 4:**
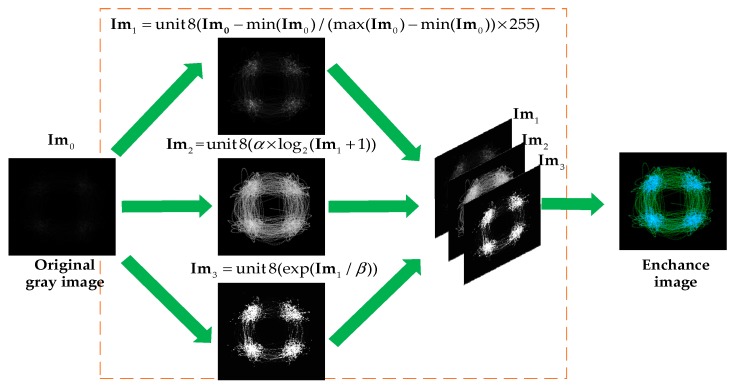
The enhancement processing of the dataset.

For modulation recognition, traditional eye diagram and vector diagram are binary images, which do not consider the signal aggregation degree at a particular location. Hence, we consider the signal aggregation degree and enhance the traditional eye diagram and vector diagram. Figure 4 presents the enhancement processing of the dataset. In our initial research, since CNNs are insensitive to edge information, the signal amplitude is quantified between [−1.05, 105] by 128 after normalizing the amplitude. Furthermore, the parameter settings are obtained by experiments. For example, we choose 800 symbols and 4 symbols as a waveform group to generate the eye diagram and the vector diagram, and related experiments will also be described in detail in subsequent chapters. Moreover, we perform the following operations on the images to make the image details more prominent, where $Im_{0}$ is the original image, $Im_{1},Im_{2},Im_{3}$ are the channels of the enhanced image and α,β are scaling factors.

(21) $$\begin{array}{l} Im_{1}=\mathrm{unit}8\left(\frac{Im_{0}-\min(Im_{0})}{\max(Im_{0})-\min(Im_{0})}\times 255\right),\\ Im_{2}=\mathrm{unit}8(\alpha \times \log2(Im_{1}+1)),\\ Im_{3}=\mathrm{unit}8(\exp(Im_{1}/\beta )) \end{array}$$

## 3. Deep Learning Framework for Signal Detection and Modulation Recognition

DL networks aim at learning different hierarchies of features from data. As one of the branches of DL techniques, the CNNs perform well in the field of image recognition. It performs feature learning via non-linear transformations implemented as a series of nested layers. Each layer consists of several kernels that perform a convolution over the input. Generally, the kernels are usually multidimensional arrays which can be updated by some algorithms [39]. Our DL framework achieves multi-signals detection and modulation recognition. We use different deep neural networks for different tasks. For signal detection, we use SSD networks. For modulation recognition, we design a multi-inputs CNNs.

### 3.1. SSD Networks for Signal Detection 

**Figure 5:**
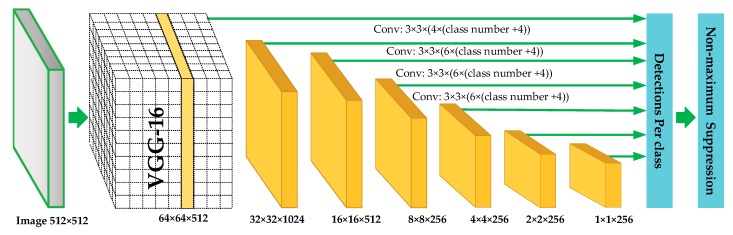
The network model for signal modulation recognition.

We use SSD networks to achieve multi-signals detection. For DL target detection techniques, the existing algorithms are mainly divided into two kinds: algorithms based on region recommendation (two-stage methods) and algorithms based on regression (one-stage methods). Regression-based algorithms include YOLO series algorithms [40,41,42,43] and SSD series algorithms [43,44], while region recommendation-based algorithms include RCNN [45], Fast RCNN [46], and Faster RCNN [47]. In our research, since the regression-based algorithms are faster than region recommendation-based algorithms, we use SSD networks as our signal detection model. SSD networks can generate a series of fixed-size borders and the possibility of the containing target in each border. Finally, the final detection and recognition results are calculated by the non-maximum suppression algorithm [48]. The structure of SSD networks is shown in Figure 5, and it can be divided into four parts.

**Part 1:** The networks for feature extraction. The initial part of SSD networks is the first layers of VGG16 network, which is used as the primary network to extract the deep features of the whole input image. Behind the primary network, the model structure is the pyramid networks, which contains a series of simple convolution layers to make feature maps smaller and smaller. With the pyramid network structure, we can get several feature maps with different scales.

**Figure 6:**
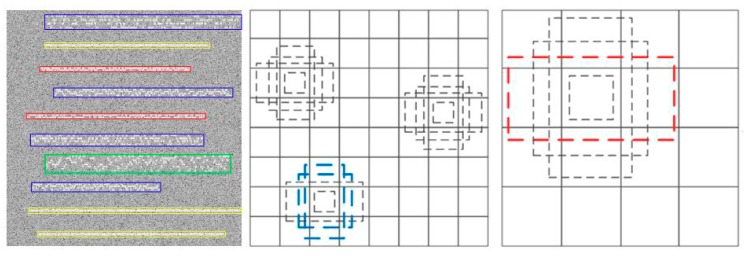
The Design of the default box.

**Part 2:** The design of the default box. In this part, we will design several feature default box for different scales of feature maps. Each feature map at the top of the VGG16 networks is associated with a set of feature default box. As shown in Figure 6, there are dotted borders at each position of 4 × 4 and 8 × 8 feature maps. These fixed-size borders are default boxes, and their scale parameters are designed by the different feature maps scales. For example, assuming that we need M feature maps to predict, the scale parameters of the default box are as follows:

(22) $$S_{k}=S_{\min}+\left(\frac{S_{\max}-S_{\min}}{m-1}\right)•\left(k-1\right),k\in \left[1,m\right]$$

where $S_{\min}$ is the bottom scale, and $S_{\max}$ is top scale. The length-width radio of feature default can be expressed as: $a_{r}\in \left\{1,2,3,1/2,1/3\right\}$. So the feature default box length is $W_{k}^{a}=S_{k}\sqrt{a_{r}}$ and the width is $H_{k}^{a}=S_{k}/\sqrt{a_{r}}$.

**Part 3:** Detection and Recognition. In this part, we can predict the target category and location. We add a set of convolution kernels behind several different scales feature maps. Using these convolution kernels, we can get a fixed set of test results. For an *m* × *n* × *p* feature maps, a small convolution kernel with 3 × 3 × *p* size is used as the fundamental prediction element. Finally, the classification probability of each feature default box and the offsets are obtained.

**Part 4:** Non-maximum suppression. In the last part, we use non-maximum suppression to select the best prediction results. For the feature default boxes that are matched by each real target border, we calculate their intersection-parallel ratios. The expression is shown as follow

(23) IoU=(A∩B)/(A∪B)

where A and B are two borders. We will select the feature default box whose IoU are greater than 0.5 as best results, and then obtains the highest confidence degree feature default box by non-maximum suppression.

In offline train stage, the whole objective optimal function of the SSD networks includes two parts: confidence loss and location loss. The expression is shown as follow

(24) $$L\left(x,c,l,g\right)=\frac{1}{N}\left(L_{conf}\left(x,c\right)+\alpha L_{loc}\left(x,l,g\right)\right)$$

where x is used to indicate whether the feature default box is a target or not. *N* is the number of the feature default boxes that are matched to real target borders. Parameter α is used to adjust the radio between $L_{conf}$ and $L_{loc}$, default α=1. $L_{conf}$ is softmax loss function. $L_{loc}$ is used to measure the performance of the boundary box prediction, and in our initial research, we use the typical $smooth_{L1}$ function to calculate

(25) $$L_{conf}\left(x,c\right)=-\sum_{i\in Pos}^{N}x_{ij}^{p}\log\left({\hat{c}}_{i}^{p}\right)-\sum_{i\in Neg}\log\left({\hat{c}}_{i}^{0}\right)\;\;,\;\;\;{\hat{c}}_{i}^{p}=\frac{\exp\left(c_{i}^{p}\right)}{\sum_{p}c_{i}^{p}}$$

(26) $$L_{loc}\left(x,l,g\right)=\sum_{i\in Pos}^{N}\sum_{m\in \left\{cx,cy,w,h\right\}}x_{ij}^{k}smooth_{L1}\left(l_{i}^{m}-{\hat{g}}_{j}^{m}\right)$$

where *Pos* and *Neg* represent all positive and negative borders, respectively. $c_{i}^{p}$ represents the confidence degree for *p*th feature default matching *i*th target. $l_{i}^{m}$ represents the prediction bias of the i-th feature default box. (cx,cy) is the box center coordinates and (w,h) is the box width and height. ${\hat{g}}_{j}^{m}$ represents the deviation between the real target border $g_{j}^{m}$ and the default box $d_{i}^{m}$. ${\hat{g}}_{j}^{m}$ is calculated as follow:${\hat{g}}_{j}^{cx}=\left(g_{j}^{cx}-d_{i}^{cx}\right)/d_{i}^{w}$, ${\hat{g}}_{j}^{cy}=\left(g_{j}^{cy}-d_{i}^{cy}\right)/d_{i}^{h}$, ${\hat{g}}_{j}^{w}=\log\left(g_{j}^{w}/d_{i}^{w}\right)$, ${\hat{g}}_{j}^{h}=\log\left(g_{j}^{h}/d_{i}^{h}\right)$.

### 3.2. Multi-Inputs CNNs for Modulation Recognition 

**Figure 7:**
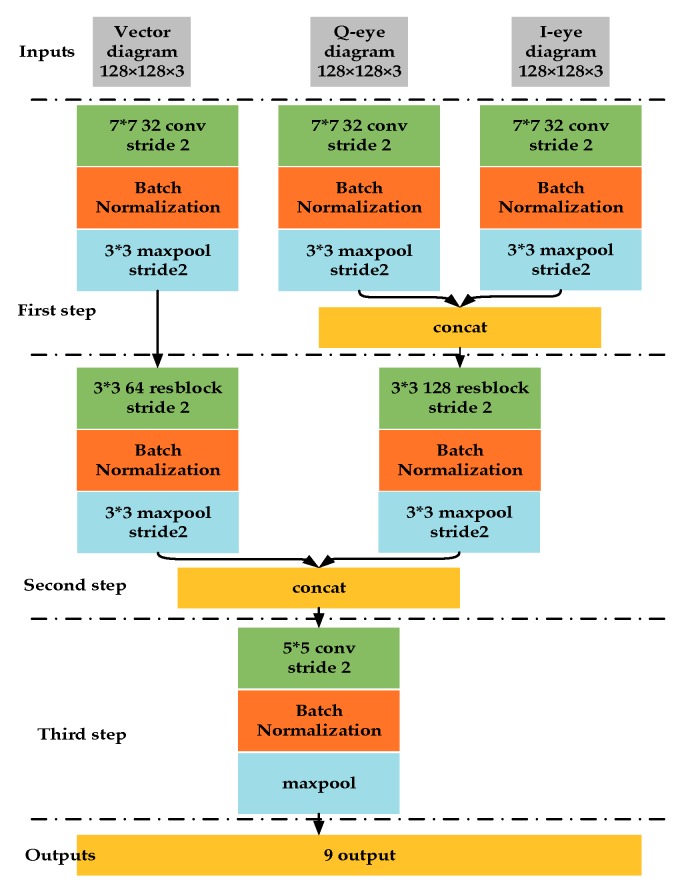
The network model for signal modulation recognition.

For signal modulation recognition task, the modulation set is {BPSK, QPSK, OQPSK, 8PSK, 16QAM, 16APSK, 32APSK, 64QAM}, because they are all belonging to amplitude-phase modulation and we cannot distinguish each other from time-frequency characteristic in SSD network. Hence, we use the eye diagram and vector diagram as the model inputs. The multi-inputs CNNs model is shown in Figure 7. The initial size of the samples is 128 × 128, and we use softmax as the output layer’s active function and relu as all other network layers’ active function.

The signal features extraction can be divided into three stages. On the first stage, we use 7 × 7 convolution kernels to convolute IQ eye diagram and vector diagram, respectively. To ensure the dynamic range unification of the feature maps, we apply the batch normalization (BN) [49] on first layer network outputs. We perform the max pooling operation on the BN feature maps. Then we connect the feature maps from IQ eye diagram inputs.

**Figure 8:**
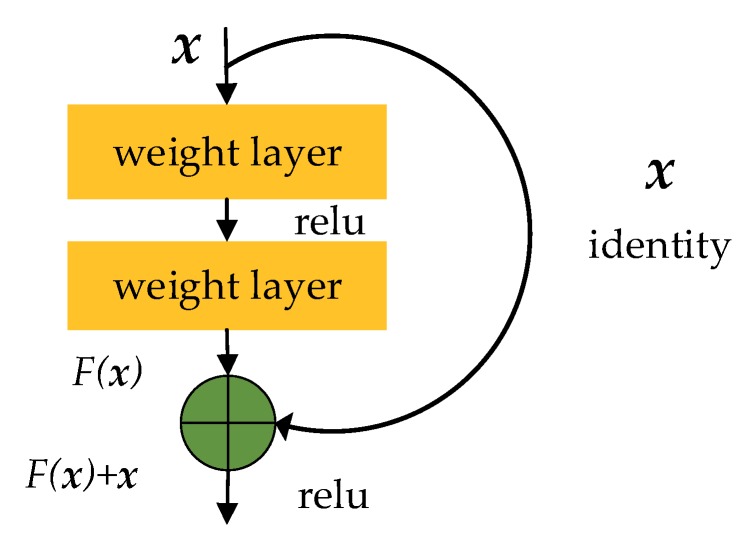
The basic structure of ResNet.

On the second signal feature extraction stage, we adopt the residual network structure to avoid the degradation caused by the network over-depth. The basic structure of ResNet [50] is shown in Figure 8. After the second feature extraction stage, each input feature maps are connected. After the batch normalization in the third stage, we directly process the feature maps by global maximum sampling to reduce the network parameters.

In the processing of network optimization, we adopt Adam algorithm [51] to solve the network parameters optimal solution. The categorical cross-entropy error is chosen as the loss function, which is represented as:

(27) $$J_{1}\left(w,b;x_{1},x_{2},x_{3},y\right)=-\sum_{i}^{N}{\left(y_{i}\right)}^{T}\log(f_{1}(x_{1,i},x_{2,i},x_{3,i};w,b))+\lambda_{1}{\sum \left\|w\right\|}^{2}$$

### 3.3. The Description for Deep Learning Framework 

**Figure 9:**
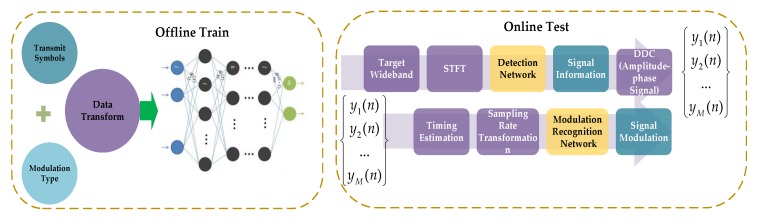
System model.

According to above introduction of the signal detection network and the modulation recognition network, we describe the use of our DL framework. Figure 9 presents the system model. The steps of the model used are as follows:

**Step 1:** We construct the signal detection network and modulation recognition network and train each network with their appropriate samples.

**Step2:** In the online testing phase, we perform STFT for the wideband signals, and use the trained SSD networks to detect the signals in the time-frequency spectrum. In this step, we can obtain the center frequency and start-stop time of each signal. And for MFSK signal, we can get its modulation format.

**Step 3:** For the amplitude-phase modulation signal, we can get the central frequency and start-stop time in step 2. With this knowledge, we filter the target signal and use the envelope spectrum to estimate the signal symbol rate. Then we down convert the signal and perform the matched filter by using the estimated symbol rate.

**Step 4:** If the timing deviation exists in the target signal, it is necessary to extract the sample value at the optimum sampling position for signal eye diagram and vector diagram. We use the non-data-aided timing estimation algorithm in [52]. The specific expression is as follows, where $L_{0}$ is the length of the signal symbols, T is the sampling period and N is the oversampling number:

(28) $$\overset{\wedge }{\tau }=\arg\left\{\sum_{k=0}^{NL_{0}-1}{\left|s\left(\frac{kT}{N}\right)\right|}^{2}e^{-j2\pi k/N}\right\}$$

**Step 5:** We alter the target signal sampling rate, and obtain the baseband signal with a maximum delay 32 sampling period. Moreover, we generate the eye-diagram and vector diagram with the processed signal.

**Step 6:** We use the trained modulation recognition network to identify the signal by its eye diagram and vector diagram. Finally, we complete the signal detection and modulation recognition.

## 4. Results

In Section 2 and Section 3, we have discussed the methods which convert complex signal samples into images without noticeable information loss and introduced the structures of our DL framework. Table 1 shows the time complexity of our DL framework on the different process, in which N is the number of signals in the wideband range. It can be seen that our framework has low time complexity due to the evolution of GPUs, which is acceptable for many practical communication systems.

We also have conducted several experiments to demonstrate the performances of the DL framework for joint signal detection and modulation recognition in wireless communication systems. Our experiments can be divided into two parts: (1) performances on multi-signals detection and (2) performances on modulation recognition. The rest of this section is organized as follows

**Multi-signals detection:** First, we show some results of our detection network, and explain the reasons for these results. Then, we evaluate the model performances from three aspects: the modulation format, carrier frequency, and start-stop time. We also compare our network with other detection networks.

**Signal modulation recognition:** We evaluate our model recognition performances on each modulated signal. We also discuss the network performances when the frequency offset exists. Meanwhile, we compare our method with traditional methods and other DL based methods. Finally, we discuss the influence of symbol and eye numbers and compare the performance between signal-input networks and multi-inputs networks.

### 4.1. Performance on Signal Detection 

**Figure 10:**
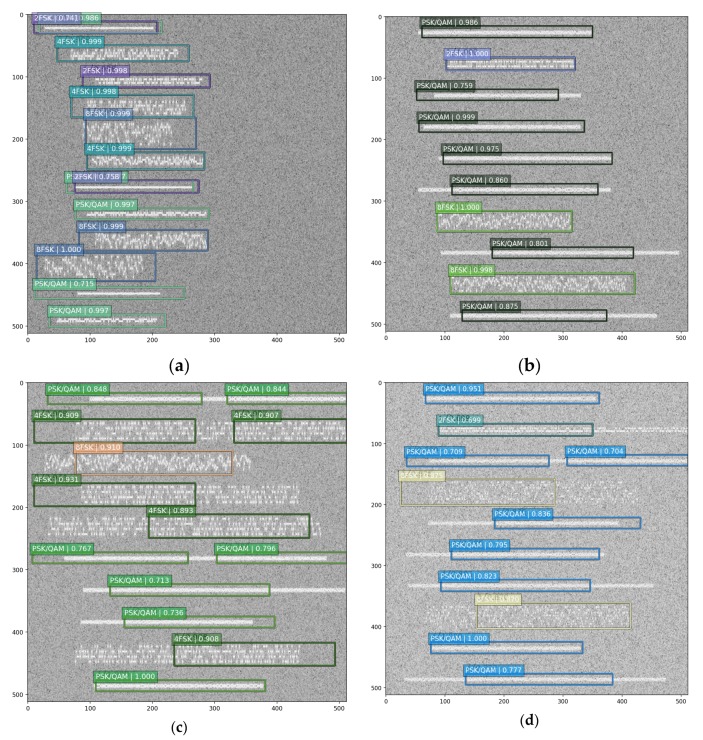

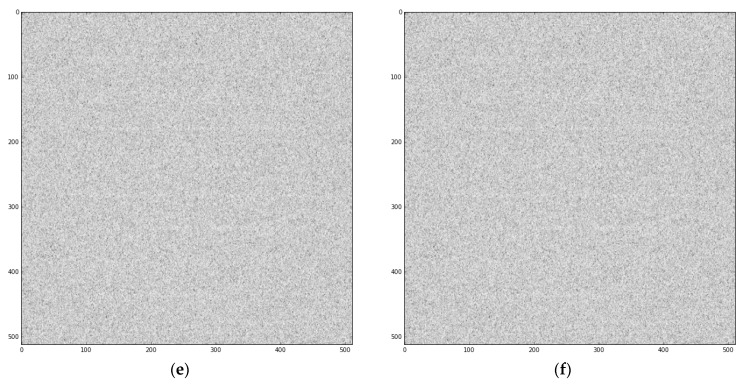
The SSD networks detection results. (**a**) ideal result 1; (**b**) ideal result 2; (**c**) not perfect result 1; (**d**) not perfect result 2; (**e**) no signal result 1; (**f**) no signal result 2.

For multi-signal detection, we need to know each signal carrier frequency, start-stop time and modulation format. Figure 10 shows some detection results from our model. From Figure 10a,b, it is indicated that our model is beneficial for multi-signals detection, and it can accurately estimate the relevant information about each signal. Moreover, our model has very a promising application prospect in engineering because it has a good visualization effect.

To some extent, our model is not perfect yet, and there are still some aspects that need to be improved. From Figure 10c,d, we can learn that once the signal length is large, the estimation of the signal start-stop time is not precise, while the estimation of the carrier frequency is precise. The cause of this phenomenon may be that the time-frequency spectrum has large deformation and extreme length-with radio, while the natural image is not. Therefore, we need to further optimize the default box in the SSD networks. Figure 10e,f show the network performance when there is no signal exists. It can be observed that the model does not produce a false alarm, which is useful in engineering.

**Figure 11:**
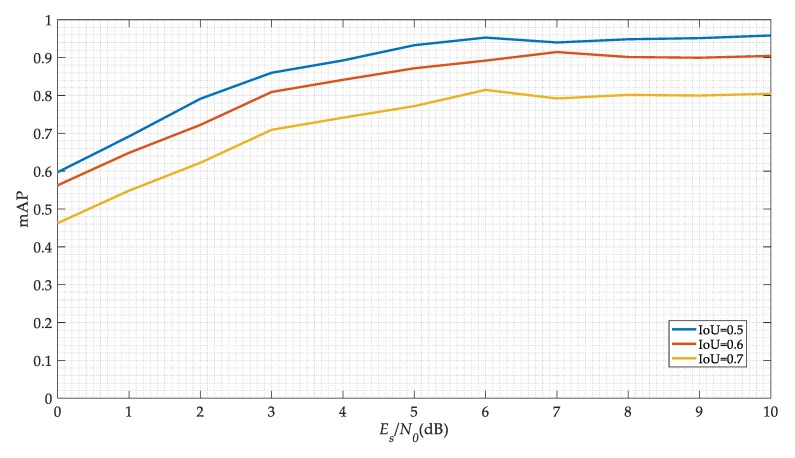
The SSD networks performances.

Figure 11 shows our model detection precision versus different SNRs. We choose the mean Average Precision (mAP) as the performance index of the model. To calculate the mAP, we need to calculate precision and recall. For calculating precision and recall, we need to identify True Positive (TP), False Positive (FP), True Negative (TN), and False Negative (FN). Recall is defined as the proportion of all positive examples ranked above a given rank. Precision is the proportion of all examples above that rank which are from positive. The Average Precision (AP) summarizes the shape of the precision/recall curve. Hence, the mAP is the mean of all the AP values across all classes as measured above. They can be calculated as follows

(29) $$\mathrm{Precision}=\frac{TP}{TP+FP},\mathrm{Recall}=\frac{TP}{TP+FN}$$

(30) $$AP=\int_{0}^{1}P(r)dr$$

(31) $$mAP=\frac{\sum_{\mathrm{num}\_\mathrm{classes}}AP_{i}}{\mathrm{num}\_\mathrm{classes}}$$

It can be deduced that with the increase of the SNR, the mAP value of the SSD network is increasing. When the SNR is 5 dB, the mAP value can reach 90% in *IoU* is 0.5. Different *IoU* thresholds can lead to different results. Although the increase of the threshold can obtain more reliable signal carrier frequency and start-stop time, it sacrifices the precision of signal detection. Besides, we can adopt some traditional methods to further estimate these signal parameters. Finally, we choose 0.5 as the threshold of *IoU*.

Once we detected the signals, we need to evaluate the precision of the estimated parameters. We use the normalized offset of the estimated and the actual parameters as the criterion of measurement. They can be presented as follows:

(32) $$\Delta f=\frac{\left|f_{pre}-f_{real}\right|}{R}$$

(33) $$\Delta t_{start}=\frac{\left|t_{pre\_start}-t_{true\_start}\right|}{T},\Delta t_{stop}=\frac{\left|t_{pre\_stop}-t_{true\_stop}\right|}{T}$$

where $f_{pre}$ is the predicted value of the carrier frequency, $f_{real}$ is the actual value of the carrier frequency, R is the symbol rate, $t_{pre\_start}$ and $t_{pre\_stop}$ are the predict values of the start and the stop time, $t_{true\_start}$ and $t_{true\_stop}$ are the actual values of the start and the stop time, and T is the signal duration. Table 2 shows the carrier frequency and the start-stop time precision when the signal is detected. It can be seen that the precision of the carrier frequency is higher than start and stop time. These phenomena are consistent with Figure 10c,d. And in future research, we need to combine the prior information of the signal to design the default boxes and the networks.

**Figure 12:**
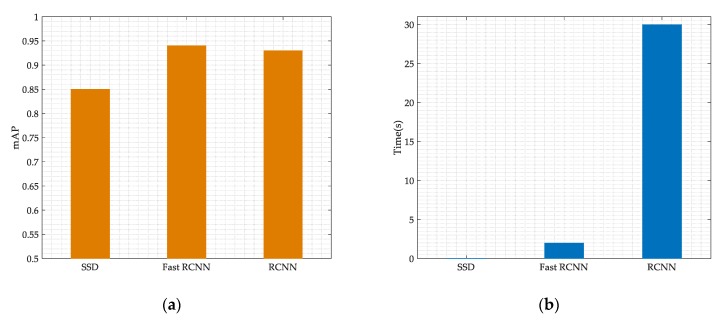
Different networks performances. (**a**) performances for mAP; (**b**) performances for time.

We also compare our model performances with the RCNN networks and the Fast RCNN. From Figure 12a, we can see that the mAP of the Fast RCNN and the RCNN is higher than the SSD networks, but the improvement is not significant. And from Figure 12b, we can infer that the SSD networks has considerable advantages in processing speed compared with the RCNN and Fast RCNN. Our model processing speed can reach 0.05 s for each time-frequency spectrum, and such a computational complexity is acceptable for many practical communications systems.

### 4.2. Performance on Modulation Recognition

**Figure 13:**
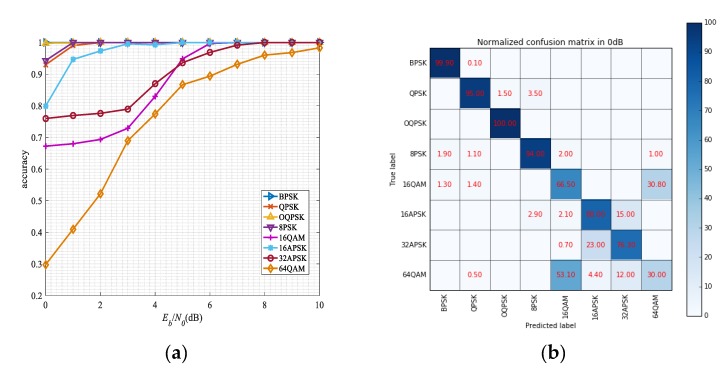

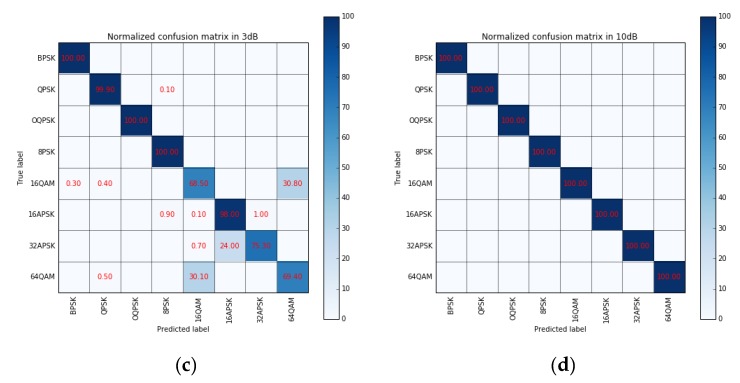
The performance of each modulation (**a**) classification accuracy for each modulation versus SNR; (**b**) normalized confusion matrix in 0 dB; (**c**) normalized confusion matrix in 3 dB; (**d**) normalized confusion matrix in 10 dB

For signal modulation recognition, we set a series of experiments to test the network performances. Figure 13a shows the recognition performances of each modulated signal under the different SNR. It can be seen that the algorithm can still achieve better performance when the SNR is very low. Because its modulation complexity, the performance of 64QAM signal is worse than other signals, but it still can achieve 94% accuracy at 7 dB. For BPSK and OQPSK signals, they have distinct visual characteristics from other modulated signals in the eye diagram and the vector diagram, which recognition accuracy can reach 100% even in 0 dB. And it is also obvious that the recognition performance of circular modulation signals {8PSK, 16APSK, 32APSK} is better than QAM modulation signal. To understand the results better, the confusion matrices in different SNR levels are presented in Figure 13b–d. It can be seen that the network shows excellent performance in discriminating BPSK, QPSK, OQPSK, 8PSK, and 16APSK. Moreover, in our experiments, it can be seen that 16QAM is more likely confused with 64QAM, while 16APSK is more likely confused with 64QAM.

For accuracy comparison, we consider four different modulation classification algorithms.

(1) Cumulant: A traditional signal processing algorithm using the fourth-order cumulant C40 as the classification statistics [19].
(2) SVM-7: An ML-based algorithm using the SVM with seven features, including three fourth-order cumulants C40, C41, and C42 and four sixth-order cumulants C60, C61, C62, and C63 [20].
(3) CNNs for IQ waveform: A DL-based algorithm using the CNNs with the signal IQ waveform [4].
(4) CNNs for constellation: An DL-based algorithm using the CNNs with the signal constellation [35].

**Figure 14:**
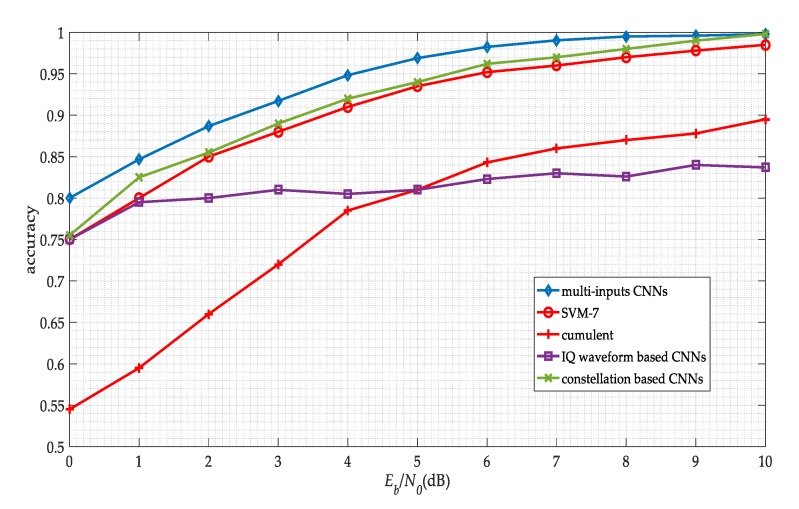
Different methods performance versus SNR.

Figure 14 presents the average classification accuracy of five algorithms versus SNR. The average accuracy is obtained by averaging the classification performance of eight modulation categories. The performance results of our algorithm outperform all other algorithms.

**Figure 15:**
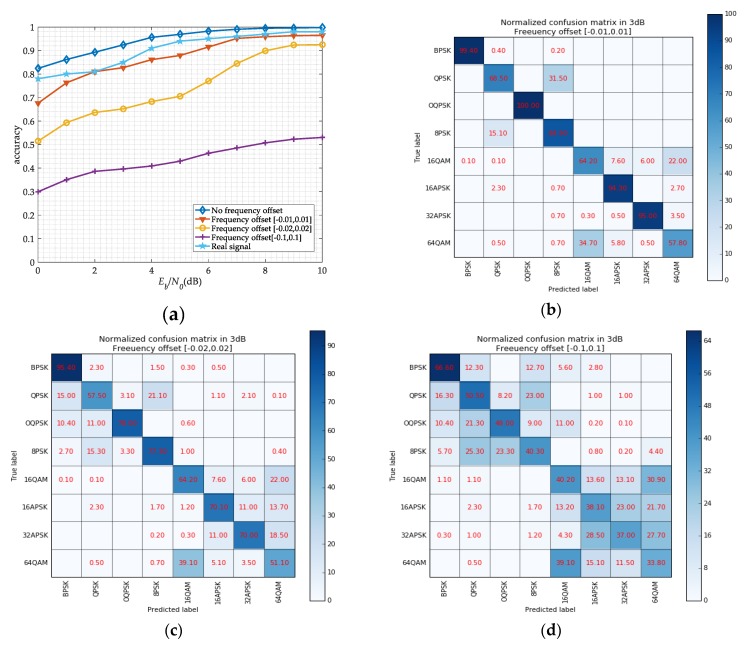
The network performance on different frequency offsets range. (**a**) classification accuracy for different frequency offset versus SNR; (**b**) normalized confusion matrix in 3 dB when the frequency offset is [−0.01, 0.01]; (**c**) normalized confusion matrix in 3dB when the frequency offset is [−0.02, 0.02]; (**d**) normalized confusion matrix in 10 dB when the frequency offset is [−0.1, 0.1].

Considering the error of the carrier frequency estimation by SSD networks and FFT in practice, we research the network recognition performance in different frequency offsets. We set a series of frequency offset for signals, and the result is shown in Figure 15. It can be seen that the recognition accuracy of the signals with a frequency offset is lower than those without frequency offset. When the signals have a large frequency offset, the network is no longer suitable. We also collect some signals from a real satellite communication system, and the real-time wireless channel is performed in the received signals. And then, we use a signal playback device, a DSP card, and PCs to simulate signal reception process. From Figure 15a, we can obtain that the recognition accuracy on real data is lower on simulated data at same SNR level. It may be due to the training data, which not consider the actual channel environment clearly. But the recognition accuracy can still reach 90% when the SNR is 4 dB. And for further research, we will make full use of the real signal to make our model more robust.

**Figure 16:**
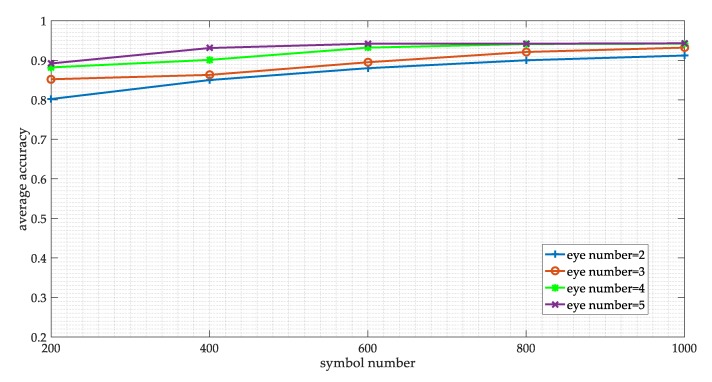
The network performance on the different sample parameters.

We also consider the influence of the symbol numbers and the eye number in eye diagram on the network performance. We obtain the best parameter settings of samples by grid search. The symbol number is set as 200, 400, 800, and 1000, respectively, while the eye number is set as 2, 3, 4, and 5. The results are shown in Figure 16. It can be seen that theses parameters do affect network performance. With the increase of symbol number and eye number, the overall accuracy of the model is gradually increasing. But we also can see that when the symbol number is 1000 and the eye number is 5, the improvement of performance is not obvious. Therefore, we finally choose 800 symbols and 4 eye numbers to generate the eye diagram and the vector diagram.

**Figure 17:**
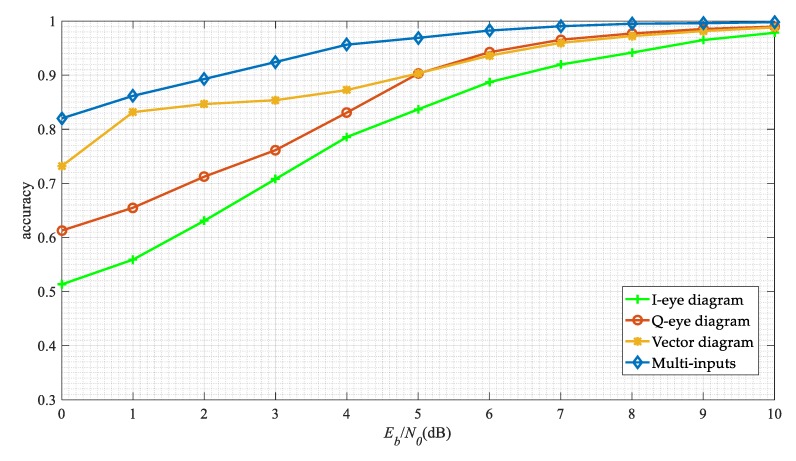
The network performance on the different input model.

Finally, we compare the performance of the single input network with the multi-inputs network in this work. The results are shown in Figure 17. The modulation recognition algorithm based on a single eye diagram has poor performance. The performance of the I-eye diagram is lower than that of the Q-eye diagram, which may be due to the setting of the initial phase in the same modulation format. And the performance of the vector diagram based method is also inferior to our method, since it does not make full use of the signal waveform information.

## 5. Conclusions and Discussions

In our research, we have demonstrated our initial efforts to establish a DL framework for multi-signals detection and modulation classification problem. In our method, the time-frequency spectrums are exploited for multi-signals detection task, while the eye-diagrams and vector diagrams are exploited for the modulation classification task. The simulation results prove that DL technologies have the ability to solve the problems in the communication field and have higher performance than other methods.

However, in the future, we will do more rigorous analysis and more comprehensive experiments. Besides, for practical use, we will collect the samples generated from the real channels, and then retrain or fine-tune the model for better performance.

## Figures and Tables

**Table 1:** The time complexity of the DL framework (ms).

Simple for Signal Detection	Signal Detection	Down Conversion	Simple for Modulation Recognition	Modulation Recognition
28.5	50.5	10.3 × *N*	20.8	10.6

**Table 2:** The offset in the estimation of the various parameters.

Offset	Carrier Frequency	Start Time	Stop Time
0 dB	2.0%	35.2%	38.6%
5 dB	1.3%	22.3%	21.4%
10 dB	0.5%	9.6%	8.7%
